# Complete Genome Sequence of *Lymantria dispar multiple nucleopolyhedrovirus* Isolated in Southwestern Poland

**DOI:** 10.1128/genomeA.01422-16

**Published:** 2016-12-22

**Authors:** Martyna Krejmer-Rabalska, Lukasz Rabalski, Iwona Skrzecz, Boguslaw Szewczyk

**Affiliations:** a Laboratory of Recombinant Vaccines, Intercollegiate Faculty of Biotechnology, University of Gdansk and Medical University of Gdansk, Gdansk, Poland; b Department of Forest Protection, Forest Research Institute, Sekocin Stary, Poland

## Abstract

We report here the complete genomic sequence of Polish alphabaculovirus isolated from dead gypsy moth caterpillars. Its genome structure and G+C content indicate that it is a *Lymantria dispar multiple nucleopolyhedrovirus* (LdMNPV) strain which possesses the shortest sequence among eight other (similar to reference sequence AF081810) LdMNPV sequences available in GenBank; it is 159,729 bp long.

## GENOME ANNOUNCEMENT

The gypsy moth, *Lymantria dispar* L., is one of the species that causes economic damage to forests and orchards. Outbreaks of gypsy moths have been observed in different parts of the world, mainly in North America. In Europe, it causes considerable damage, mainly in oak stands (1). Since the late 1950s, scientists have attempted to use a nucleopolyhedrovirus (NPV) associated with *L. dispar*, *Lymantria dispar multiple nucleopolyhedrovirus* (LdMNPV), as a biological pesticide.

An alphabaculovirus presented in this report was isolated from dead gypsy moth caterpillars found in 2004 in Rudy Raciborskie, Silesia, Poland. Its complete genome is the ninth isolate of LdMNPV species deposited in the GenBank database. There is also one alphabaculovirus isolate called LdMNPV-BNP (GenBank accession no. KU377538) found in dead gypsy moth caterpillars in Biebrzanski National Park, Poland. However, this isolate shows more genetic similarities to *Lymantria monacha* baculovirus than any other LdMNPVs sequenced to date (2). The isolate reported in this paper and strain LdMNPV-3054 from Spain are the only two of European origin (3) for which complete genomic sequences are available.

The genome of LdMNPV_RR01 strain is 159,729 bp long, with a G+C content of 57.5%, which is similar (57.3 to 57.5%) to that of other complete genomes of LdMNPV strains available in GenBank (GenBank accession numbers AF081810, KF695050, KM386655, KP027546, KT626570, KU862282, KT626571, and KT626572). The predicted number of encoded putative open reading frames (ORFs) is 166. All core genes were found in the nucleotide genomic sequence. The number of baculovirus-repeated ORF (*bro*) genes is 15 (*bro-a* to *bro-o*). All LdMNPV genomes are collinear. The structure of the complete genome is similar to that of others, with minor differences, such as the lack of one viral enhancing factor (*vef-1*), which is equivalent to the deletion of 2.4 kbp. The same situation occurs in a geographically distant strain from Japan, LdMNPV-3041. The number of homologous repeated sequences (*hrs*) is 12 and meets standards for LdMNPV repeated regions (from 12 to 13).

DNA extraction was performed using a MagAttract high-molecular-weight (HMW) DNA kit (Qiagen), according to the manufacturer’s protocol. DNA sequencing was done at the Medical University of Gdansk in Poland, using MiSeq (Illumina) and version 3 sequencing chemistry. For genomic library preparation, the Nextera XT DNA sample prep kit (Illumina) was used. During the machine run, paired reads of the target size 2 × 300 bp were generated. After a trimming procedure by removing low-quality reads and contaminants, 4,093,620 reads with at least Q30 (90.1%) were used to produce a single circular contig by *de novo* assembly using Geneious 9 (Biomatters). After remapping of all reads to the contig, the mean coverage was 4,700×, with standard deviation of 1,397.3×. Regions with repeated and unsure sequences were confirmed by PCR amplification and Sanger sequencing. ORFs were identified by Glimmer3 (at least 150 nucleotides [nt] long) and then manually checked and compared with other LdMNPV strains. The *polh* gene was annotated as a first ORF. Repeated regions were found using sequences characteristic for LdMNPV (4).

### Accession number(s).

The genome sequence of LdMNPV_RR01 has been deposited in GenBank under the accession no. KX618634.

## References

[1] Feltwell J 1989 Gypsies attack forest—or is it acid rain? Br J Entomol Nat Hist 2:77–79.

[2] Rabalski L, Krejmer-Rabalska M, Skrzecz I, Wasag B, Szewczyk B 2016 An alphabaculovirus isolated from dead *Lymantria dispar* larvae shows high genetic similarity to baculovirus previously isolated from *Lymantria monacha*—an example of adaptation to a new host. J Invertebr Pathol 139:56–66. doi:10.1016/j.jip.2016.07.011. 27451947

[3] Harrison RL, Rowley DL, Keena MA 2016 Geographic isolates of *Lymantria dispar* multiple nucleopolyhedrovirus: genome sequence analysis and pathogenicity against European and Asian gypsy moth strains. J Invertebr Pathol 137:10–22. doi:10.1016/j.jip.2016.03.014. 27090923

[4] Kuzio J, Pearson MN, Harwood SH, Funk CJ, Evans JT, Slavicek JM, Rohrmann GF 1999 Sequence and analysis of the genome of a baculovirus pathogenic for *Lymantria dispar*. Virology 253:17–34. doi:10.1006/viro.1998.9469. 9887315

